# Vertical and Horizontal Genetic Connectivity in *Chromis verater*, an Endemic Damselfish Found on Shallow and Mesophotic Reefs in the Hawaiian Archipelago and Adjacent Johnston Atoll

**DOI:** 10.1371/journal.pone.0115493

**Published:** 2014-12-17

**Authors:** Kimberly A. Tenggardjaja, Brian W. Bowen, Giacomo Bernardi

**Affiliations:** 1 Department of Ecology and Evolutionary Biology, University of California Santa Cruz, Santa Cruz, California, United States of America; 2 Hawaii Institute of Marine Biology, University of Hawaii, Kaneohe, Hawaii, United States of America; Kunming Institute of Zoology, Chinese Academy of Sciences, China

## Abstract

Understanding vertical and horizontal connectivity is a major priority in research on mesophotic coral ecosystems (30–150 m). However, horizontal connectivity has been the focus of few studies, and data on vertical connectivity are limited to sessile benthic mesophotic organisms. Here we present patterns of vertical and horizontal connectivity in the Hawaiian Islands-Johnston Atoll endemic threespot damselfish, *Chromis verater*, based on 319 shallow specimens and 153 deep specimens. The mtDNA markers cytochrome *b* and control region were sequenced to analyze genetic structure: 1) between shallow (<30 m) and mesophotic (30–150 m) populations and 2) across the species' geographic range. Additionally, the nuclear markers rhodopsin and internal transcribed spacer 2 of ribosomal DNA were sequenced to assess connectivity between shallow and mesophotic populations. There was no significant genetic differentiation by depth, indicating high levels of vertical connectivity between shallow and deep aggregates of *C. verater*. Consequently, shallow and deep samples were combined by location for analyses of horizontal connectivity. We detected low but significant population structure across the Hawaiian Archipelago (overall cytochrome *b*: Φ_ST_ = 0.009, *P* = 0.020; control region: Φ_ST_ = 0.012, *P* = 0.009) and a larger break between the archipelago and Johnston Atoll (cytochrome *b*: Φ_ST_ = 0.068, *P*<0.001; control region: Φ_ST_ = 0.116, *P*<0.001). The population structure within the archipelago was driven by samples from the island of Hawaii at the southeast end of the chain and Lisianski in the middle of the archipelago. The lack of vertical genetic structure supports the refugia hypothesis that deep reefs may constitute a population reservoir for species depleted in shallow reef habitats. These findings represent the first connectivity study on a mobile organism that spans shallow and mesophotic depths and provide a reference point for future connectivity studies on mesophotic fishes.

## Introduction

The majority of coral reef ecosystems studied to date occur at depths shallower than 30 m, yet zooxanthellate corals can extend to depths of over 150 m [1]. Mesophotic coral ecosystems (MCEs or “deep reefs”) make up this “twilight zone” of 30–150 m [2], [3]. The establishment of MCEs depends on multiple factors, including light penetration, water temperature, and substrate availability [3]. In some areas where shallow reefs thrive, strong thermoclines can prevent the development of mesophotic reefs [4], and the depth at which light is not sufficient to support zooxanthellae defines the lower limit of MCEs [1], [3]. The upper boundary of mesophotic reefs is based on the depth limit of conventional SCUBA diving (30–40 m) [1].

Studies on vertical and horizontal connectivity have been highlighted as priorities in MCE research [3], [5]–[7]. Most connectivity studies on shallow-reef organisms have assessed horizontal connectivity across the range of a given species, whereas vertical connectivity refers to the movement of individuals between depth zones. One of the major motivations for understanding vertical connectivity is evaluating the possibility that mesophotic reefs can seed shallow reefs. As postulated in the “deep reef refugia” hypothesis, MCEs may act as a reproductive source that restocks depleted shallow reefs or as a haven where populations can escape adverse conditions [4], [6], [8]. Given the vulnerability of MCEs to anthropogenic effects that also plague shallow reefs [6], an additional motivation for studying connectivity in these ecosystems is to prevent the loss of potentially unique genetic diversity.

Vertical connectivity has been the primary emphasis of mesophotic genetic studies to date, with less focus on horizontal connectivity. Kahng et al. [7] summarized current knowledge about connectivity in MCEs. First, there is a growing number of mesophotic studies that demonstrate limited vertical connectivity in sessile benthic organisms [9]–[13]. This genetic structure may be the result of adaptations to unique environmental conditions at different depths [1], [7], [12], [14], [15]. For mobile marine organisms, no generalized patterns are known for vertical connectivity because few studies of this nature exist. A second pattern in MCE connectivity studies is that high levels of horizontal connectivity may be common for mesophotic organisms [9], [10], [16]–[18].

**Figure 1:**
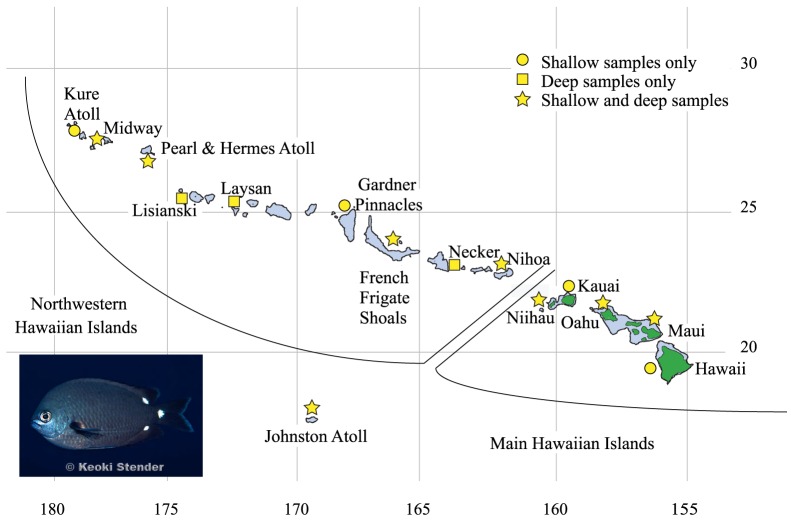
Map of collection locations. Collection locations for *Chromis verater*. Shapes indicate whether shallow (circle), mesophotic (square), or both shallow and mesophotic (star) specimens were collected at the location. (Photo credit: Keoki Stender, www.marinelifephotography.com).

Here we apply mitochondrial and nuclear markers to assess connectivity in the threespot chromis, *Chromis verater* [19], which inhabits shallow and deep reefs. The pelagic larval duration of *C. verater* is not known but has been postulated to last as long as three months [20]. The depth range of this species is from 7 m to a maximum recorded depth of 199 m [21], and it is usually sparse in shallow water and abundant at depths greater than 18 m [20], [22]. The abundance of juveniles in deeper water indicates that *C. verater* may recruit in deeper habitats, migrating into shallower water later in life [20], [23]. This planktivorous damselfish is endemic to the Hawaiian Archipelago and to adjacent Johnston Atoll, which is located about 860 km south of the archipelago (Fig. 1). The Hawaiian Archipelago, which comprises the eight main Hawaiian Islands (MHI) and the nine Northwestern Hawaiian Islands (NWHI) (Fig. 1), is one of the few areas in the Pacific where progress is being made in MCE exploration. These ecosystems exhibit a patchy distribution throughout the archipelago, with better developed and deeper MCEs occurring near the southern end [24], [25]. Large fish communities have been observed on some mesophotic reefs but, for unknown reasons, are absent from others [26]. Surveys of MCEs in the NWHI revealed that 46% of fishes on mesophotic reefs are endemic species, in comparison to 21% endemism on shallow reefs in this region [27], [28]. Thus, MCEs harbor fish communities that overlap with those on shallow reefs but also have unique attributes [29], [30].

Our study addresses two primary issues: 1) vertical connectivity between shallow and mesophotic populations of *C. verater* and 2) horizontal connectivity across mesophotic populations and also across the geographic range of this species. With respect to vertical connectivity, we predict exchange between shallow reefs and MCEs based on the abundance of juveniles at depth. Regarding horizontal connectivity, most reef fishes show no structure across the Hawaiian Archipelago, but the exceptions tend to be endemics [31], [32]. In particular, a previous study on the endemic damselfishes *Dascyllus albisella* and *Stegastes marginatus* demonstrated genetic structure across the Hawaiian Archipelago and between the archipelago and Johnston Atoll [33].

## Materials and Methods

### Tissue collection and ethics statement

Across the species range in the Hawaiian Archipelago and Johnston Atoll, 319 shallow and 153 mesophotic *C. verater* specimens (fin clips) were collected. While the majority of specimens were adults, 1 shallow and 58 deep individuals were juveniles, based on field observations of body size. Collections at 12 shallow sites were made with pole spears or hand nets with SCUBA or while snorkeling (Fig. 1). Collections at 11 mesophotic sites were made using open-circuit technical diving, rebreather diving, and submersibles, and many of the mesophotic specimens (herein referred to as “deep specimens”) were collected during research expeditions to explore MCEs in the Hawaiian Archipelago and Johnston Atoll (Fig. 1). Although data are unavailable for exact depths at which most specimens were collected, shallow specimens were collected above 30 m, and deep specimens were collected at depths below 30 m with a greatest depth of 113 m. All tissue collections were made under permits PMNM-2007-032, PMNM-2008-046, PMNM-2009-032L, PMNM-2009-044, PMNM-2011-025, and PMNM-2012-045, issued by the National Oceanic and Atmospheric Administration, the U.S. Fish and Wildlife Service, and the State of Hawaii Division of Aquatic Resources to BWB at the Hawaii Institute of Marine Biology. Sampling protocols for this study were approved by the Institutional Animal Care and Use Committees at the University of California Santa Cruz and the University of Hawaii.

### DNA extraction, marker amplification, and sequencing

Tissue specimens were preserved in salt-saturated water with 20% DMSO [34], and genomic DNA was extracted using the HotSHOT method [35]. Individuals were amplified for two mitochondrial markers: cytochrome *b* (cyt*b*) and control region (CR). Cyt*b* was amplified with primers GLUDG-5′ [36] and H16460 (http://nmg.si.edu/bermlab.htm). CR was amplified with primers Pro-L [37] and CR-E [38]. These markers were chosen so that our results could be compared to previous studies and in case the more variable non-coding CR would resolve patterns not detected in cyt*b*.

To verify that observed patterns were not restricted to the mitochondrial genome, subsets of the shallow (*N* = 49) and mesophotic (*N* = 45) specimens from the Hawaiian Archipelago were amplified for two nuclear markers: rhodopsin and internal transcribed spacer 2 of ribosomal DNA (ITS2). Rhodopsin was amplified according to published nested amplification protocols, using RHO-30F and RHO-319R for the first set of primers and Rod-F2x and Rod-R4n in the second [39]. ITS2 was amplified following published protocols, using primers 5.8sr and 28s [40].

Polymerase chain reactions (PCRs) were performed in 14 µl reactions containing 1 µl of diluted DNA extract (one part DNA to 49 parts of nanopure water), 0.29 µl of each 10 µM primer, 7.14 µl of premixed PCR solution MangoMix™ (Bioline Inc., Springfield, NJ, USA), and 5.28 µl of nanopure water. PCR amplification of cytb consisted of an initial denaturation at 94°C for 3 min, followed by 35 cycles of 45 s at 94°C, 1 min 15 s at 50°C, and 1 min 15 s at 72°C, with a final extension for 5 min at 72°C. PCR amplification of CR consisted of an initial denaturation at 94°C for 5 min, followed by 35 cycles of 30 s at 94°C, at 49°C, and at 72°C, with a final extension for 7 min at 72°C. After purification of PCR products following the manufacturer's protocol (Applied Biosystems, Foster City, CA, USA), sequencing was performed with the forward PCR primers on an ABI 3730xl DNA analyzer (Applied Biosystems, Foster City, CA, USA) at the University of California Berkeley's DNA Sequencing Facility. Sequences were aligned and edited using Geneious R6 (Biomatters, LTD, Auckland, NZ). Alignments of cytb and rhodopsin were unambiguous, while CR and ITS2 each contained multiple indels, which varied from 1–2 bp (CR) and 1–28 bp (ITS2) in length. For the nuclear markers, IUPAC ambiguity codes were used to score heterozygous individuals. Unique haplotypes for each marker were identified in Arlequin and were uploaded to GenBank (accession numbers KP183329-KP183902).

### Genetic diversity and population structure analyses

Haplotype diversity (*h*) and nucleotide diversity (*π*) were calculated in Arlequin 3.5 [41]. Population structure was analyzed in terms of vertical connectivity and horizontal connectivity, using analyses of molecular variance (AMOVAs) and population pairwise Φ_ST_ comparisons in Arlequin. The Φ_ST_ fixation index incorporates genetic distance and ranges from 0 to 1, with low values indicating a lack of genetic structure and high values indicating genetic differentiation. Significance of pairwise Φ_ST_ comparisons and AMOVA calculations was tested with 10,000 permutations, and to correct for multiple comparisons, a modified false discovery rate method was implemented [42]. We determined the best model of sequence evolution for each marker in jModeltest2 [43], [44]. Since the models identified by the Akaike information criterion were not available in Arlequin, we selected the Tamura-Nei model as it was the most similar [45]. Because Midway deep (*N* = 2) and Necker deep (*N* = 1) had small sample sizes, they were included in adjacent populations of Pearl and Hermes deep and French Frigate Shoals deep respectively for most population genetic analyses, after establishing that their haplotypes were closely related to those at these adjacent sites. Parsimony-based haplotype networks for each marker were constructed in R using haploNet in the package pegas 0.5–1 [46]. Haplotype frequencies used in these networks were calculated in Arlequin.

For comparisons within the Hawaiian Archipelago, we wanted to rule out the possibility that the large sample size of shallow specimens (*N* = 296) was overwhelming population structure due to the mesophotic specimens (*N* = 129). To accomplish this, we ran AMOVAs with equal sample sizes for the shallow and mesophotic specimens. The shallow dataset was randomly subsampled for 129 individuals to match the number of mesophotic specimens, and subsampling was replicated ten times. Then for both mitochondrial markers, AMOVAs were run with the full set of Hawaiian mesophotic specimens and each of the shallow subsample sets to determine whether there was significant genetic structure between shallow and deep.

To avoid making *a priori* assumptions about the possible locations of genetic barriers, we used the computational geometry approach in Barrier 2.2 [47] to visualize where genetic barriers are located in geographic space. Genetic barriers represent changes in genetic composition between sample sites. The software identifies where genetic barriers are located geographically by using Voronoi tessellation and Delaunay triangulation, implementing Monmonier's maximum-difference algorithm to compare a distance matrix (e.g. matrix of pairwise population Φ_ST_ values) with a matrix of geographic distances. *A posteriori* AMOVAs subsequently were performed on population groupings inferred by Barrier output.

To determine whether any observed genetic structure was due to isolation by distance, Mantel tests were performed to test for a correlation between genetic distance and geographic distance. Mantel tests were run in the vegan package in R with 10,000 permutations, using matrices of pairwise Φ_ST_ values and geographic distances as calculated by the Geographic Distance Matrix Generator [48], [49].

### Migration analyses

To infer directionality of gene flow, we used Migrate 3.6.4 [50], [51], which estimates coalescence-based average migration rates. We concatenated mitochondrial markers for these analyses on the advice of the software author (P. Beerli, pers. comm.), allowing for site rate variation in four categories. Since Migrate employs the F84 mutation model, the gamma shape alpha parameter for this model was estimated in PAUP 4.0, as well as the transition-transversion ratio [52]. Estimates of *θ* (4*N*_e_µ) and *M* (*m*/µ) were generated using slice sampling. We employed the recommended Bayesian inference search strategy with a single 500,000 or 700,000 step chain, discarding the first 25% as burn-in [51]. Initial runs utilized default uniform priors and the unrestricted migration model. Posterior distributions of *θ* and *M* informed priors for subsequent runs. Once parameters were optimized, runs were replicated three times, utilizing four short heated chains. We estimated the number of migrants between regions per generation (*Nm*) by multiplying estimates of *M* and *θ* of the destination population. Migrate was run to test for both horizontal connectivity and vertical connectivity. For horizontal connectivity, the program was run among Johnston Atoll, the NWHI, and the MHI, using 47 individuals randomly sampled from each of those groups. For vertical connectivity, the program was run for individual sampling locations where shallow and mesophotic specimens were available and had *N*>10 for each depth zone. Also, the program was run with two groups: one with all shallow Hawaiian individuals and one with all deep Hawaiian individuals. Since the shallow Hawaiian Archipelago and deep Hawaiian Archipelago runs did not have unimodal normally distributed posterior distributions, we do not present these data. Though our Migrate estimates are based on one locus, they are still useful for finding relative differences within this dataset.

## Results

A total of 719 bp of cyt*b* and 394 bp of CR were resolved for 319 shallow and 153 mesophotic *C. verater* specimens, including those from Johnston Atoll. Summary statistics for number of haplotypes (*H*), haplotype diversity (*h*), and nucleotide diversity (*π*) are listed in Table 1. Nucleotide diversity across shallow sites was similar to that across mesophotic sites for both markers (Table 1). Overall haplotype diversity was very high with *h* = 0.9041 to 0.9066 for cyt*b* and *h* = 0.9994 to 0.9997 for CR (Table 1). For cyt*b*, haplotype diversity values for shallow Johnston Atoll (*h* = 0.6245) and deep Johnston Atoll (*h* = 0.7645) were lower than that of any site in the Hawaiian Archipelago (*h* = 0.8182–0.9722). Nearly every CR sequence was a unique haplotype, so haplotype diversity was even higher for this marker and had a narrower range across the various sites (*h* = 0.9833–1.0000). Haplotype diversity across shallow sites was similar to that across mesophotic sites for both markers (Table 1). When we controlled for sample size at each location, there was no significant difference in haplotype diversity (data not shown).

**Table 1:** MtDNA molecular diversity indices for shallow and mesophotic samples of *Chromis verater*.

			Cyt*b*						CR					
	*N*		*H*		*π*		*h*		H		π		*h*	
*Sample location*	shallow	deep	shallow	deep	shallow	deep	shallow	deep	shallow	deep	shallow	deep	shallow	deep
**Hawaiian Archipelago**														
Kure	6	-	6	-	0.0026±0.0020	-	1.0000±0.0962	-	6	-	0.0695±0.0412	-	1.0000±0.0962	-
Midway	34	-	18	-	0.0035±0.0021	-	0.9091±0.0353	-	34	-	0.0828±0.0412	-	1.0000±0.0071	-
Pearl and Hermes	30	15	18	10	0.0036±0.0022	0.0027±0.0018	0.9402±0.0269	0.9238±0.0530	30	14	0.0850±0.0425	0.0890±0.0461	1.0000±0.0086	0.9905±0.0281
Lisianski	-	5	-	4	-	0.0022±0.0018	-	0.9000±0.1610	-	5	-	0.0713±0.0443	-	1.0000±0.1265
Laysan	-	16	-	11	-	0.0029±0.0019	-	0.9083±0.0633	-	16	-	0.0839±0.0433	-	1.0000±0.0221
Gardner Pinnacles	12	-	6	-	0.0021±0.0015	-	0.8182±0.0840	-	12	-	0.0912±0.0482	-	1.0000±0.0340	-
French Frigate Shoals	30	9	13	8	0.0026±0.0017	0.0035±0.0023	0.8713±0.0395	0.9722±0.0640	29	9	0.0893±0.0446	0.0889±0.0487	0.9977±0.0094	1.0000±0.0524
Nihoa	32	4	18	4	0.0036±0.0022	0.0044±0.0034	0.9435±0.0231	1.0000±0.1768	32	4	0.0889±0.0442	0.0959±0.0637	1.0000±0.0078	1.0000±0.1768
Niihau	45	22	26	16	0.0040±0.0024	0.0036±0.0023	0.9424±0.0224	0.9610±0.0260	41	22	0.0888±0.0438	0.0885±0.0447	0.9960±0.0057	1.0000±0.0137
Kauai	30	-	21	-	0.0035±0.0022	-	0.9494±0.0276	-	27	-	0.0844±0.0422	-	0.9931±0.0105	-
Oahu	31	41	16	22	0.0027±0.0017	0.0032±0.0020	0.8903±0.0396	0.8915±0.0416	28	41	0.0871±0.0434	0.0921±0.0455	0.9935±0.0100	1.0000±0.0054
Maui	16	17	10	10	0.0027±0.0018	0.0033±0.0021	0.9000±0.0619	0.9191±0.0438	14	17	0.0822±0.0425	0.0881±0.0452	0.9833±0.0278	1.0000±0.0202
Island of Hawaii	30	-	14	-	0.0028±0.0018	-	0.8851±0.0425	-	30	-	0.0862±0.0430	-	1.0000±0.0086	-
*All of Hawaiian Archipelago*	296	129	83	49	0.0032±0.0020	0.0032±0.0020	0.9131±0.0102	0.9155±0.0170	271	128	0.0792±0.0384	0.0808±0.0393	0.9993±0.0004	0.9999±0.0010
**Johnston Atoll**														
Johnston Atoll	23	24	7	8	0.0021±0.0015	0.0029±0.0019	0.6245±0.1096	0.7645±0.0765	21	22	0.0616±0.0314	0.0651±0.0330	0.9921±0.0154	0.9928±0.0144

Number of individuals (*N*), number of haplotypes (*H*), nucleotide diversity (*π*), and haplotype diversity (*h*) are listed for cyt*b* and CR. Because Midway deep (*N* = 2) and Necker deep (*N* = 1) had small sample sizes, they were included in adjacent populations of Pearl and Hermes deep and French Frigate Shoals deep respectively for most population genetic analyses.

**Figure 2:**
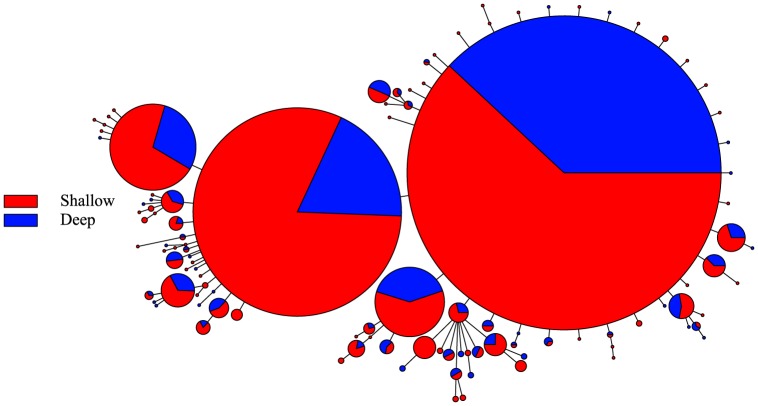
Cyt*b* haplotype network for *Chromis verater.* Parsimony-based network using cyt*b* sequence data and color-coded according to depth at which specimens were collected.

**Figure 3:**
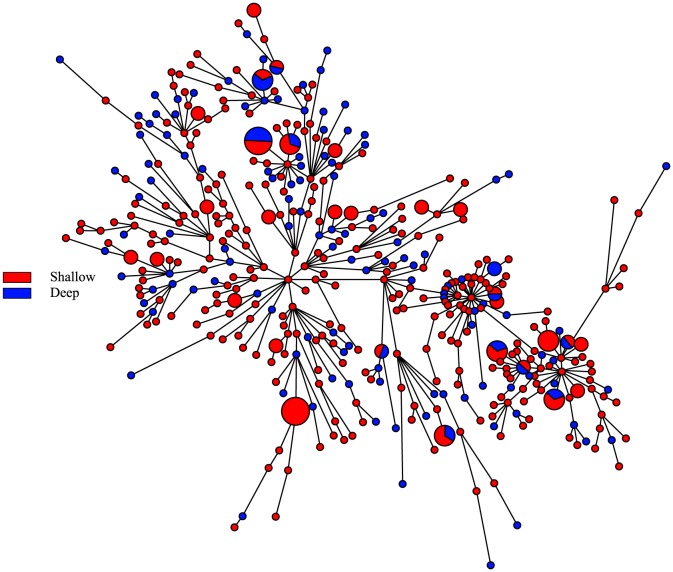
CR haplotype network for *Chromis verater.* Parsimony-based network using CR sequence data and color-coded according to depth at which specimens were collected.

The haplotype networks for cyt*b* and CR in *C. verater* do not illustrate clustering of haplotypes by depth (Fig. 2 and Fig. 3). In the network for cyt*b*, the three most common haplotypes were detected in both shallow and mesophotic individuals. Since nearly each CR sequence constituted a unique haplotype, the shape of this network is very different from that for cyt*b*. Nevertheless, there seems to be abundant intermixing of shallow and mesophotic specimens. In the Supporting Information, the same haplotype networks are presented but are color-coded according to geographic sampling location (S1 Figure and S2 Figure). Overall, haplotypes do not appear to group by geographic location, except for some clustering of Johnston Atoll haplotypes in the CR haplotype network.

A total of 442 bp of rhodopsin and 401 bp of ITS2 were sequenced for 49 shallow and 45 mesophotic *C. verater* specimens from the Hawaiian Archipelago. Summary statistics for number of haplotypes (*H*), haplotype diversity (*h*), and nucleotide diversity (*π*) for the nuclear markers are listed in S1 Table. Nucleotide diversity across shallow sites was higher than that across mesophotic sites for both nuclear markers (S1 Table). Haplotype diversity across shallow sites was similar to that across mesophotic sites.

**Figure 4:**
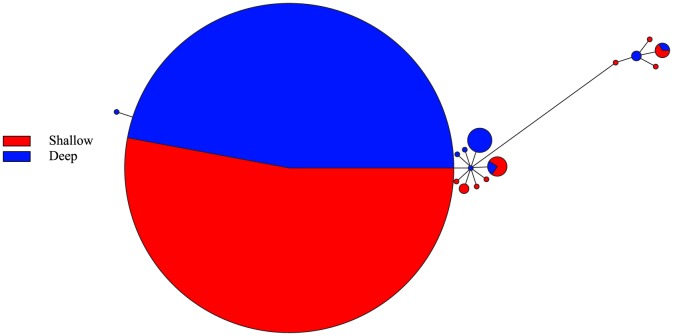
ITS2 haplotype network for *Chromis verater*. Parsimony-based network using ITS2 sequence data for subsample of 94 specimens and color-coded according to depth at which specimens were collected.

**Figure 5:**
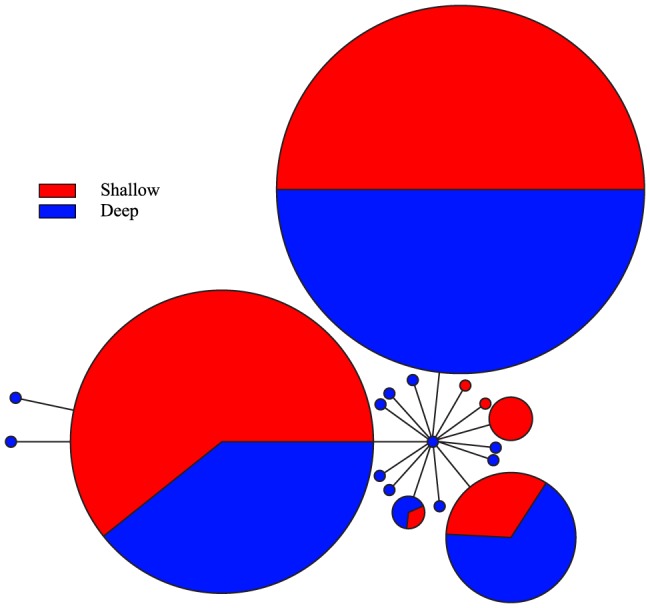
Rhodopsin haplotype network for *Chromis verater*. Parsimony-based network using rhodopsin sequence data for subsample of 94 specimens and color-coded according to depth at which specimens were collected.

Similar to the haplotype networks for the mtDNA markers, the networks for ITS2 and rhodopsin do not show clustering of haplotypes by depth (Fig. 4 and Fig. 5). The network for rhodopsin is dominated by three common haplotypes, and the ITS2 network has one common haplotype. When color-coded by sampling location, in the network for ITS2, a few haplotypes comprised of individuals from Kauai, Lisianski, and Maui deep are divergent from the other haplotypes by 12 mutations (S3 Figure). Nonetheless, the haplotype networks do not show clustering by geographic location (S3 Figure and S4 Figure).

### Vertical connectivity

To determine if there was significant genetic differentiation by depth in *C. verater*, first we ran an AMOVA separating all of the specimens into two groups: shallow and mesophotic. Neither cyt*b* nor CR demonstrated significant genetic structure between the shallow and mesophotic groups (cyt*b*: Φ_ST_ = 0.003, *P* = 0.079; CR: Φ_ST_ = 0.003, *P* = 0.103) (Table 2). In addition, when Johnston Atoll individuals were removed from the analysis, there was no evidence of significant structure by depth across the Hawaiian Archipelago (cyt*b*: Φ_ST_ = 0.001, *P* = 0.217; CR: Φ_ST_ = 0.000, *P* = 0.366). Likewise, AMOVAs indicated no significant genetic structure between shallow and deep populations for either nuclear marker (rhodopsin: Φ_ST_ = −0.018, *P* = 0.779; ITS2: Φ_ST_ = −0.009, *P* = 0.482). For the mtDNA markers, none of the individual locations that had shallow and mesophotic individuals demonstrated significant population structure across depth (Table 2).

**Table 2:** Analyses of molecular variance (AMOVAs) for vertical connectivity in *Chromis verater*, using different groupings of populations.

	Cyt*b*			CR		
Groupings	% variation within populations	Φ_ST_	*P* value	% variation within populations	Φ_ST_	*P* value
**Johnston Atoll and Hawaiian Archipelago**						
All shallow/all deep	99.71	0.003	0.079	99.74	0.003	0.103
Shallow Johnston Atoll/deep Johnston Atoll	100	−0.020	0.705	100	−0.025	0.941
**Hawaiian Archipelago**						
All shallow/all deep	99.87	0.001	0.217	100	0.000	0.366
Shallow Pearl and Hermes/deep Pearl and Hermes	100	−0.007	0.550	100	−0.001	0.372
Shallow French Frigate Shoals/deep French Frigate Shoals	100	−0.029	0.768	100	−0.041	0.952
Shallow Nihoa/deep Nihoa	100	−0.039	0.635	100	−0.038	0.674
Shallow Niihau/deep Niihau	100	−0.007	0.693	100	−0.018	0.980
Shallow Oahu/deep Oahu	100	−0.011	0.864	100	−0.009	0.800
Shallow Maui/deep Maui	97.97	0.020	0.181	98.63	0.014	0.210

Percent variation within populations (% variation), fixation indices (Φ_ST_), and associated *P* values are listed. “/” is used to separate different groupings of sampling locations.

For the mtDNA comparisons within the Hawaiian Archipelago where we employed the subsampling procedure for the shallow specimens, nine out of ten runs showed no evidence of population structure by depth (Table 3). One run indicated very weak structure that was nearly significant for cyt*b* (Φ_ST_ = 0.005, *P* = 0.061) and significant for control region (Φ_ST_ = 0.007, *P* = 0.044). Since there was a lack of significant vertical genetic structure in the majority of these runs, we did not perform additional subsampling runs.

**Table 3:** AMOVAs for vertical connectivity runs with sets of shallow subsamples (*N* = 129) in *Chromis verater*.

	Cytb			CR		
Subsample	% variation within populations	Φ_ST_	*P* value	% variation within populations	Φ_ST_	*P* value
1	99.95	0.001	0.333	100	−0.002	0.729
2	99.46	0.005	0.061	99.29	**0.007**	**0.044**
3	99.82	0.002	0.214	100	−0.001	0.571
4	100	−0.004	0.977	100	−0.002	0.767
5	100	−0.002	0.645	100	−0.003	0.842
6	100	0.000	0.396	100	−0.001	0.484
7	99.89	0.001	0.276	100	−0.002	0.766
8	99.87	0.001	0.255	99.82	0.002	0.212
9	100	−0.003	0.916	100	−0.002	0.653
10	100	0.000	0.436	100	−0.002	0.627

AMOVAs were run with specimens divided into shallow individuals and deep individuals. Percent variation within populations (% variation), fixation indices (Φ_ST_), and associated *P* values are listed. Bold values are significant (*P*<0.05).

Effective migration rates using Bayesian methods revealed that migration between shallow and deep populations varied by sampling location (Table 4). At Johnston Atoll, there was a subtle bias in migration from shallow to deep (*Nm* = 305.27, 95% CI = 37.96–945.22) than from deep to shallow (*Nm* = 251.99, 95% CI = 17.27–853.03). Likewise, the estimated number of migrants per generation was much higher from shallow to deep for Maui and Oahu. Conversely, at Pearl and Hermes and Niihau, the migration rate was higher from deep to shallow. No general pattern was apparent from these data.

**Table 4:** Migration rate estimates (*Nm*) into destination populations for *Chromis verater*.

	Estimated number of migrants per generation		
	2.5% percentile	Mean	97.5% percentile
**Vertical connectivity analyses**			
Pearl and Hermes: shallow into deep	0	11.05	58.80
Pearl and Hermes: deep into shallow	65.60	593.19	1925.86
Niihau: shallow into deep*	0	149.79	244.26
Niihau: deep into shallow*	0	228.22	230.58
Oahu: shallow into deep*	386.96	2305.59	5893.00
Oahu: deep into shallow*	0	6.80	38.63
Maui: shallow into deep	42.34	484.54	1618.41
Maui: deep into shallow	0	6.57	37.57
Johnston Atoll: shallow into deep	37.96	305.27	945.22
Johnston Atoll: deep into shallow	17.27	251.99	853.03
**Horizontal connectivity analyses**			
MHI into NWHI*	0	2914.15	21010.14
NWHI into MHI*	14.22	34329.53	131805.70
MHI into Johnston Atoll	0	1.15	97.07
Johnston Atoll into MHI	0	1179.08	8223.50
NWHI into Johnston Atoll	0	1.33	97.07
Johnston Atoll into NWHI	0	252.77	3074.67

Mean number of migrants per generation into destination populations and 95% confidence intervals are reported. Vertical connectivity analyses are estimates of migration from shallow into deep and from deep into shallow for five sampling locations that had both shallow and mesophotic specimens available and *N*>10 for each depth zone. Horizontal connectivity analyses are estimates of migration between the main Hawaiian Islands (MHI), the Northwestern Hawaiian Islands (NWHI), and Johnston Atoll.

*Some parameters may not have converged, which would have affected these migration rate estimates.

### Horizontal connectivity

When we performed an AMOVA using all Johnston Atoll and Hawaiian locations without separating shallow and mesophotic individuals, weak yet significant population structure was detected for both cyt*b* and CR (cyt*b*: Φ_ST_ = 0.023, *P*<0.001; for CR: Φ_ST_ = 0.036, *P*<0.001) (Table 5). Johnston Atoll was driving this structure, as it was significantly different in pairwise comparisons from almost all locations in the Hawaiian Archipelago (Table 6). Also, Barrier identified a genetic break between Johnston Atoll and the Hawaiian Archipelago. AMOVAs run with individuals grouped into these two regions confirmed that this break was significant (cyt*b*: Φ_ST_ = 0.068, *P*<0.001; for CR: Φ_ST_ = 0.116, *P*<0.001) (Table 5).

**Table 5:** AMOVAs for horizontal connectivity in *Chromis verater*, using different groupings of populations.

	Cyt*b*			CR		
Groupings	% variation within populations	Φ_ST_	*P* value	% variation within populations	Φ_ST_	*P* value
**Johnston Atoll and Hawaiian Archipelago**						
All (shallow and deep combined per sampling location)	97.68	**0.023**	**<0.001**	97.06	**0.036**	**<0.001**
All deep	96.54	**0.035**	**0.002**	96.84	**0.032**	**0.003**
All shallow	98.88	**0.011**	**0.018**	97.10	**0.029**	**<0.001**
All Johnston Atoll/all Hawaiian Archipelago	93.21	**0.068**	**<0.001**	88.44	**0.116**	**<0.001**
Shallow Johnston Atoll/shallow Hawaiian Archipelago	95.24	**0.048**	**0.002**	88.08	**0.119**	**<0.001**
Deep Johnston Atoll/deep Hawaiian Archipelago	92.92	**0.071**	**<0.001**	90.88	**0.091**	**<0.001**
**Hawaiian Archipelago**						
All (shallow and deep combined per sampling location)	99.07	**0.009**	**0.020**	98.85	**0.012**	**0.009**
All deep	98.69	0.013	0.127	100.00	−0.001	0.500
All shallow	99.68	0.003	0.247	99.05	0.010	0.055
Island of Hawaii/rest of archipelago	97.89	**0.021**	**0.019**	96.48	**0.035**	**0.005**

Percent variation within populations (% variation), fixation indices (Φ_ST_), and associated *P* values are listed. “/” is used to separate different groupings of sampling locations. Bold values are significant (*P* <0.05).

**Table 6:** Population pairwise Φ_ST_ values for *Chromis verater*.

Location	1	2	3	4	5	6	7	8	9	10	11	12	13	14
1. Kure	-	−0.026	−0.032	0.012	−0.017	−0.036	−0.011	0.027	−0.033	−0.033	−0.021	−0.024	0.018	0.199*
2. Midway	−0.056	-	0.010	−0.008	−0.013	−0.007	0.024	0.055*	0.000	−0.012	0.000	−0.006	0.064*	0.147*
3. Pearl and Hermes	−0.057	−0.010	-	0.033	0.013	−0.014	−0.003	0.013	−0.004	−0.001	0.002	0.003	0.016	0.137*
4. Lisianski	**0.210**	**0.116**	**0.144**	-	−0.019	0.008	0.040	0.084	0.024	−0.006	0.026	0.005	**0.108**	0.192*
5. Laysan	−0.014	−0.009	0.004	0.053	-	0.006	0.033	**0.071**	0.004	−0.002	0.004	−0.006	0.079	0.129*
6. Gardner Pinnacles	−0.005	−0.010	−0.019	0.297*	0.031	-	−0.016	0.012	−0.006	−0.016	−0.005	−0.012	0.005	0.159*
7. French Frigate Shoals	−0.014	0.012	−0.003	0.239*	**0.045**	−0.037	-	0.005	0.002	0.012	0.009	0.008	0.010	0.126*
8. Nihoa	−0.018	**0.025**	0.013	**0.141**	0.025	−0.014	0.010	-	**0.028**	**0.045**	0.039*	0.050*	−0.005	0.180*
9. Niihau	−0.052	0.002	−0.005	0.143*	0.012	−0.017	−0.002	0.009	-	−0.002	−0.001	0.001	**0.034**	0.129*
10. Kauai	−0.049	−0.008	−0.010	0.158*	0.006	−0.015	0.002	**0.028**	−0.002	-	−0.004	−0.007	**0.056**	0.137*
11. Oahu	−0.048	0.000	−0.006	0.186*	0.015	−0.010	0.004	0.027*	−0.001	−0.008	-	−0.011	0.052*	0.105*
12. Maui	−0.054	−0.005	−0.009	**0.157**	0.014	0.000	0.010	**0.035**	0.003	−0.006	−0.007	-	0.062*	0.109*
13. Island of Hawaii	0.010	**0.042**	0.022	0.245*	**0.069**	−0.023	−0.006	−0.001	0.014	**0.038**	**0.037**	**0.041**	-	0.214*
14. Johnston Atoll	0.030	0.047*	0.070*	**0.258**	**0.059**	**0.094**	0.105*	0.129*	0.074*	**0.038**	0.065*	0.059*	0.156*	-

Cyt*b* below the diagonal and CR above. Bold denotes significant values (*P*<0.05) and * denotes significance after application of the false discovery rate (*P*≤0.01).

Specifically to test for connectivity across mesophotic sites, an AMOVA was performed across all Johnston Atoll and Hawaiian mesophotic sites, revealing low but significant structure (cyt*b*: Φ_ST_ = 0.035, *P* = 0.002; CR: Φ_ST_ = 0.032, *P* = 0.003) (Table 5). Again, Johnston Atoll specimens were driving this genetic structure. When the analysis was run without Johnston Atoll, the population structure was not significant across the Hawaiian mesophotic sites (cyt*b*: Φ_ST_ = 0.013, *P* = 0.127; CR: Φ_ST_ = −0.001, *P* = 0.500).

To determine if there was population structure across the Hawaiian Archipelago, we ran AMOVAs with Johnston Atoll removed from the analyses. When all Hawaiian populations were included without distinguishing between shallow and deep specimens, the overall population structure was weak but significant (cyt*b*: Φ_ST_ = 0.009, *P* = 0.020; CR: Φ_ST_ = 0.012, *P* = 0.009) (Table 5). Population pairwise tests shed light on which populations are driving this signal (Table 6). For cyt*b*, Lisianski was significantly different in all pairwise comparisons, except with the adjacent location at Laysan. When Lisianski was excluded from the AMOVA, the overall population structure across the archipelago was no longer significant for cyt*b* (Φ_ST_ = 0.004, *P* = 0.117) but remained significant for CR (Φ_ST_ = 0.011, *P* = 0.011). For both cyt*b* and CR, the island of Hawaii was significantly different in at least half of the comparisons (6 for cyt*b*; 7 for CR). In the analysis of the archipelago, Barrier identified a genetic break between the island of Hawaii and the rest of the Hawaiian populations. Grouping individuals into these two regions in an AMOVA confirmed a significant break (cyt*b*: Φ_ST_ = 0.021, *P* = 0.019; CR: Φ_ST_ = 0.035, *P* = 0.005) (Table 5).

Mantel tests were performed combining shallow and deep specimens by location both with Johnston Atoll individuals and with only Hawaiian locations. There was no evidence for isolation by distance across the Hawaiian Archipelago (cyt*b*: r = 0.022, *P* = 0.400; CR: r = 0.010, *P* = 0.427). When Johnston Atoll was included in the analysis, there was no significant correlation between Φ_ST_ and geographic distance (cyt*b*: r = 0.031, *P* = 0.384; CR: r = 0.089, *P* = 0.243).

Coalescent-based estimates of migration provided insight into the direction of gene flow between Johnston Atoll, the NWHI, and the MHI (Table 4). With respect to migration between Johnston Atoll and regions of the Hawaiian Archipelago, migration estimates were much higher toward the archipelago from Johnston Atoll (to MHI: *Nm* = 1179.08, 95% CI = 0.00–8223.50; to NWHI: *Nm* = 252.77, 95% CI = 0.00–3074.67) than from the archipelago to Johnston Atoll. Within the Hawaiian Archipelago, there was greater gene flow from the NWHI into the MHI (*Nm* = 34329.53, 95% CI = 14.22–131805.70) than from the MHI into the NWHI.

## Discussion

This study represents the first attempt to assess: 1) horizontal connectivity across mesophotic populations and 2) vertical connectivity between shallow and mesophotic reefs in a species of reef fish. We acknowledge the shortcomings of low mesophotic sample sizes and uneven geographic sampling, which are due to the difficulty of collecting specimens at mesophotic depths. It would be premature to use these data on *C. verater* to make broad generalizations about connectivity patterns in mesophotic fishes, and caution should be exercised in extending these results to other types of fishes that occur at depth. Nevertheless, the results presented here portray a reef fish species that spans shallow and mesophotic depths and provide an initial reference point for understanding connectivity in mobile mesophotic organisms.

### Vertical connectivity

Using the mitochondrial markers cyt*b* and CR, we found high levels of genetic connectivity between shallow and mesophotic populations of *C. verater* in the Hawaiian Archipelago. At individual locations where shallow and mesophotic individuals had been collected, there was no significant genetic differentiation by depth. For the Hawaiian Archipelago, the large number of shallow specimens (*N* = 296) was not obscuring a signal of genetic structure from the mesophotic specimens (*N* = 129). When analyses were run with equivalent sample sizes of shallow and mesophotic individuals, nine out of ten runs exhibited high levels of vertical connectivity. We dismissed the possibility that this trend was limited to the mitochondrial genome by sequencing a subset of specimens for nuclear markers rhodopsin and ITS2, which also failed to demonstrate genetic differentiation by depth. For individual sampling locations where shallow and deep individuals were collected, coalescent-based Migrate estimates revealed that migration tended to be biased in one direction, but the direction varied by sampling location, with greater migration from shallow to deep at Johnston Atoll, Maui, and Oahu and greater migration from deep to shallow at Pearl and Hermes and Niihau.

Explicit collection depths were not available for most specimens, raising the possibility that the lack of vertical genetic structure is due to uncertainty in categorizing specimens as shallow or mesophotic. However, most mesophotic specimens were collected during expeditions to explore deep reefs (50–150 m) with open-circuit technical diving, rebreather diving, or submersibles, so we believe that this potential for error is minimal. To address this concern, future connectivity studies that span shallow and mesophotic reefs may want to consider a sampling approach that targets three depth categories, such as shallow (<20 m), middle (20–40 m), and deep (40+ m). This would allow for comparison of the shallowest and deepest individuals, as well as a separate comparison of specimens that were collected near the threshold depth of 30 m. Execution of such a sampling strategy would be more difficult but could perhaps reveal fine-scale vertical connectivity patterns. Additionally, future studies may benefit from the application of more variable loci.

The lack of genetic structure between shallow and mesophotic *C. verater* contrasts with a number of mesophotic studies that demonstrate limited vertical connectivity in sedentary benthic organisms [7]. Multiple coral species exhibit genetic partitioning by depth, with the deepest individuals often segregating as the most genetically distinct [9]–[13]. This is likely the result of adaptation to environmental conditions specific to different depths [14], [15]. While corals must rely on their gametes for dispersal potential, fishes also have the ability to disperse as juveniles/adults, which may contribute to the vertical genetic homogeneity in *C. verater*. Furthermore, *C. verater* is suspected to have a life history trait that would explain connectivity between populations at different depths: *C. verater* larvae may settle on deep reefs, gradually migrating inshore with age [20].

With respect to the “deep reef refugia” hypothesis, the extensive vertical connectivity revealed by our results implies that mesophotic populations of *C. verater* are capable of replenishing shallow populations. This is supported by the migration estimates for Pearl and Hermes and Niihau, which indicate greater gene flow from deep to shallow populations. However, other locations showed that the opposite is also true for *C. verater*, with higher migration estimates from shallow to deep. So far, it appears that the ability for mesophotic populations to serve as “deep reef refugia” varies by site and by organism. For example, at Scott Reef in northwestern Australia, there was evidence of migration from deep (31–43 m) to shallow (25–27 m) colonies in the scleractinian coral *Seriatopora hystrix*. Meanwhile, there was no evidence to support the “deep reef refugia” hypothesis at Yonge reef in northeastern Australia, where this species did not exhibit migration from deep to shallow colonies [13]. In the Caribbean scleractinian coral *Montastraea cavernosa*, regional differences in patterns of vertical connectivity suggest that the likelihood for deep reefs to restock shallow reefs varies among and within geographic locations due to local hydrology [53]. Additional connectivity studies will elucidate whether these varied patterns extend to other mesophotic fishes as well.

### Horizontal connectivity across mesophotic reefs

For the analyses of horizontal connectivity using the mitochondrial markers, we made no distinction between shallow and mesophotic individuals, combining them per location. Nevertheless, an AMOVA run with only the mesophotic individuals did not indicate significant genetic structure across the Hawaiian Archipelago. The only anomaly with the mesophotic specimens was that the Lisianski population was significantly different in most pairwise comparisons for cyt*b*. Since there were no shallow individuals available for this location, it is not certain that this pattern is unique to mesophotic individuals.

There are limited genetic connectivity studies on mesophotic reef fishes with which to compare the results from *C. verater*. Genetic studies on the submesophotic Hawaiian Grouper *Hyporthodus quernus* and deepwater snappers [17], [32], [54], [55] revealed little genetic structure within the Hawaiian Archipelago, but these are not really equivalent comparisons for *C. verater* because those species are not tightly associated with MCE habitat. Studies on mesophotic corals reveal mixed patterns of horizontal connectivity. The coral *S. hystrix* exhibited more genetic structure between depths than horizontally across geographic locations [9]. In contrast, the mesophotic red coral *Corallium rubrum* demonstrated significant geographic genetic differentiation at multiple spatial scales, from tens of meters to hundreds of kilometers, illustrating limited horizontal connectivity [16]. Similarly, *M. cavernosa* demonstrated low horizontal connectivity as well as genetic differentiation by depth [10]. Since there was no evidence of genetic structure between depths in our study, the results from our phylogeographic analyses should reflect connectivity patterns across mesophotic reefs.

### Phylogeography of a Hawaiian Islands-Johnston Atoll endemic

When shallow and mesophotic individuals were combined, the results indicate limitations to horizontal connectivity across the 860 km that separate Johnston Atoll and the Hawaiian Archipelago (cyt*b*: Φ_ST_ = 0.068, *P*<0.001; CR: Φ_ST_ = 0.116, *P*<0.001). This trend remained significant regardless of whether shallow, mesophotic, or shallow/mesophotic specimens were analyzed. The Johnston Atoll population was significantly differentiated from almost all of the Hawaiian locations in pairwise comparisons for both mitochondrial markers. Furthermore, Migrate analyses demonstrated that gene flow was biased from Johnston Atoll toward the Hawaiian Islands.

The genetic distinctiveness of Johnston Atoll populations in comparison to the Hawaiian Islands has been documented previously [56], [57], including in *Dascyllus albisella*, another Hawaiian Islands-Johnston Atoll endemic damselfish [33]. Based on oceanographic models, there are potential dispersal corridors between Johnston Atoll and French Frigate Shoals in the mid-archipelago and between Johnston Atoll and Kauai in the MHI [58]. Johnston Atoll has been implicated as a stepping stone for colonization of the Hawaiian Archipelago [32], [59]–[61]. Conversely, for some species, Johnston Atoll seems to act more as an outpost for Hawaiian fauna [56], [57]. Though higher gene flow from Johnston Atoll to the Hawaiian Archipelago supports the stepping stone possibility, the population structure analyses indicate that Johnston Atoll is serving as an outpost for *C. verater*. Cyt*b* and CR haplotype diversities for *C. verater* at Johnston Atoll are lower than at any Hawaiian site. Lower genetic diversity could be an artifact of a founder event, in which Johnston Atoll was colonized by a few individuals, or it could be indicative of a smaller population.

Within the Hawaiian Archipelago, Barrier identified a significant genetic break between the island of Hawaii and the rest of the archipelago, and this was supported by low but significant AMOVAs with both mitochondrial markers. This genetic break is concordant with one of the strongest marine barriers previously identified in the Hawaiian Archipelago and is believed to be based on oceanographic conditions [62]. The Alenuihaha Channel that separates Maui and the island of Hawaii is regarded by native navigators as some of the most dangerous waters in the archipelago, as indicated by the name which translates into “I'll-end-you-ha-ha”. Winds channeled off the adjacent peaks of Maui (3000 m high) and the island of Hawaii (4100 m high) can be five times stronger than winds outside of the channel. The prevailing northeasterly trade winds produce cyclonic mesoscale eddies on the lee side of the island of Hawaii [63] that have been reported to last as long as 60 days, sufficient for many reef fish larvae to complete their pelagic stage [64]. Christie et al. [65] posit that active behavior mechanisms allow larvae of the yellow tang, *Zebrasoma flavescens*, to extricate themselves from eddies and settle back on reefs. In that same study, virtual drifters released at 30 m depth in ocean circulation models stayed closer to the island of Hawaii than drifters released at sea surface level. If *C. verater* larvae recruit to deep reefs (>30 m) as hypothesized, then they may complete their pelagic larval duration in these eddies, retained near the island of Hawaii. Notably, this explanation does not apply to Lisianski, the only other location in the archipelago to show a low but significant level of population differentiation. Lisianski, a small (1.5 km^2^) flat outpost of coral reef habitat, lies 1676 km northwest of Oahu (Fig. 1). Explanations of genetic differentiation due to genetic drift or population size seem unlikely since the large Neva Shoals coral habitat (980 km^2^) lies directly southeast of Lisianski. Instead, it is more likely that oceanographic conditions unknown to us are driving this trend at Lisianski.

### Conclusions and implications for conservation

This genetic survey of *C. verater*, a reef fish occupying both shallow and mesophotic reefs, constitutes the first glimpse of connectivity patterns for mobile organisms that inhabit MCEs. This species exhibits high connectivity between shallow (<30 m) and mesophotic reefs (>30 m) in the Hawaiian Archipelago and Johnston Atoll, while maintaining weak population structure across this range. This dichotomy between vertical and horizontal connectivity provides an interesting perspective on dispersal in endemic species. The restricted range sizes of endemic reef fishes is thought to be a reflection of their limited dispersal abilities [31]. The lack of genetic structure between shallow and mesophotic specimens in our dataset indicates that dispersal abilities do not limit *C. verater* in terms of vertical connectivity, a scale of 7–113 m in this study. However, vertical connectivity is on a much smaller scale than horizontal connectivity, which show some limitations within the Hawaiian Archipelago (2600 km) and between the archipelago and Johnston Atoll (860 km).

Our study on connectivity in *C. verater* is relevant to emerging conservation issues for MCEs. Biodiversity hotspots are a focus for conservation efforts, and endemic species are a large component of regional biodiversity [66]–[68]. In the NWHI, endemic reef fishes were over twice as abundant on MCEs as on shallow reefs, enhancing the argument for protecting MCEs as potential biodiversity hotspots [28]. Another motivation for protection of mesophotic reefs is that they may provide critical nursery habitat for reef fishes [24]. Our results indicate that there is a lot of exchange between shallow and mesophotic populations of *C. verater*, highlighting the link between these deep reefs and other parts of coral reef ecosystems. The high levels of vertical connectivity observed in our study lend support to the argument that MCEs serve an important ecological role as habitat and refugia for populations that may be depleted in shallow habitats. Marine protected areas that encompass shallow habitats and adjacent mesophotic reefs will allow for the protection of refugia that may replenish shallow reefs.

## Supporting Information

S1 Figure **Cyt** ***b*** **haplotype network for** ***Chromis verater*** **.** Parsimony-based network using cyt*b* sequence data and color-coded by sampling location.
(EPS)

S2 Figure **CR haplotype network for** ***Chromis verater*** **.** Parsimony-based network using CR sequence data and color-coded by sampling location.
(EPS)

S3 Figure **ITS2 haplotype network for Chromis verater.** Parsimony-based network using ITS2 sequence data for subsample of 94 specimens and color-coded by sampling location.
(EPS)

S4 Figure **Rhodopsin haplotype network for Chromis verater.** Parsimony-based network using rhodopsin sequence data for subsample of 94 specimens and color-coded by sampling location.
(EPS)

S1 Table **Nuclear molecular diversity indices for shallow and mesophotic samples of Chromis verater.** Number of individuals (N), number of haplotypes (H), nucleotide diversity (π), and haplotype diversity (h) are listed for subsample of 94 individuals sequenced for rhodopsin and ITS2.
(PDF)
