# Supplementary material for: Vertical and Horizontal Genetic Connectivity in Chromis verater, an Endemic Damselfish Found on Shallow and Mesophotic Reefs in the Hawaiian Archipelago and Adjacent Johnston Atoll
Source: PLoS One. 2014 Dec 17;9(12):e115493. doi: 10.1371/journal.pone.0115493 (PMC4269425; doi:10.1371/journal.pone.0115493)
Supplement: S1 Table — Nuclear molecular diversity indices for shallow and mesophotic samples of Chromis verater. Number of individuals (N), number of haplotypes (H), nucleotide diversity (π), and haplotype diversity (h) are listed for subsample of 94 individuals sequenced for rhodopsin and ITS2. (PDF) [file pone.0115493.s005.pdf]

Table S1. Nuclear molecular diversity indices for shallow and mesophotic samples of *Chromis verater*.

			**Rhodopsin**						**ITS2**					
	N		***H***		***π***		***h***		***H***		***π***		***h***	
Sample location	**shallow**	**deep**	**shallow**	**deep**	**shallow**	**deep**	**shallow**	**deep**	**shallow**	**deep**	**shallow**	**deep**	**shallow**	**deep**
Midway	-	2	-	1	-	0.0000 ± 0.0000	-	0.0000 ± 0.0000	-	1	-	0.0000 ± 0.0000	-	0.0000 ± 0.0000
Pearl and Hermes	-	7	-	4	-	0.0005 ± 0.0008	-	0.8095 ± 0.1298	-	2	-	0.0012 ± 0.0013	-	0.4762 ± 0.1713
Lisianski	-	3	-	1	-	0.0000 ± 0.0000	-	0.0000 ± 0.0000	-	2	-	0.1318 ± 0.0993	-	0.6667 ± 0.3143
Laysan	-	11	-	3	-	0.0005 ± 0.0007	-	0.6364 ± 0.0895	-	4	-	0.0069 ± 0.0044	-	0.6000 ± 0.1539
Niihau	8	5	4	3	0.0006 ± 0.0009	0.0000 ± 0.0000	0.7500 ± 0.1391	0.7000 ± 0.2184	1	2	0.0000 ± 0.0000	0.0000 ± 0.0000	0.0000 ± 0.0000	0.4000 ± 0.2373
Kauai	16	-	8	-	0.0015 ± 0.0014	-	0.8417 ± 0.0748	-	6	-	0.0967 ± 0.0496	-	0.6167 ± 0.1347	-
Oahu	12	4	8	2	0.0014 ± 0.0014	0.0000 ± 0.0000	0.8939 ± 0.0777	0.5000 ± 0.2652	2	2	0.0000 ± 0.0000	0.0000 ± 0.0000	0.1667 ± 0.1343	0.5000 ± 0.2652
Maui	-	13	-	5	-	0.0004 ± 0.0007	-	0.8333 ± 0.0597	-	5	-	0.0566 ± 0.0299	-	0.5385 ± 0.1611
Island of Hawaii	13	-	4	-	0.0005 ± 0.0007	-	0.7564 ± 0.0698	-	6	-	0.01071 ± 0.0063	-	0.7179 ± 0.1279	-
All of Hawaiian Archipelago	49	45	15	7	0.0011 ± 0.0011	0.0003 ± 0.0006	0.8129 ± 0.0378	0.7131 ± 0.0418	10	9	0.0409 ± 0.0205	0.0269 ± 0.0138	0.4600 ± 0.0884	0.4879 ± 0.0887

Number of individuals (*N*), number of haplotypes (*H*), nucleotide diversity (*π*), and haplotype diversity (*h*) are listed for subsample of 94 individuals sequenced for rhodopsin and ITS2.
